# Recruitment of Young Gay, Bisexual, and Other Men Who Have Sex With Men for a Web-Based Human Papillomavirus Vaccination Intervention: Differences in Participant Characteristics and Study Engagement by Recruitment Source in a Randomized Controlled Trial

**DOI:** 10.2196/64668

**Published:** 2025-01-03

**Authors:** Daniel J Marshall, Amy L Gower, Mira L Katz, José A Bauermeister, Abigail B Shoben, Paul L Reiter

**Affiliations:** 1 College of Public Health The Ohio State University Columbus, OH United States; 2 Division of General Pediatrics and Adolescent Health Department of Pediatrics University of Minnesota Medical School Minneapolis, MN United States; 3 Comprehensive Cancer Center The Ohio State University Columbus, OH United States; 4 Department of Family and Community Health School of Nursing University of Pennsylvania Philadelphia, PA United States

**Keywords:** study recruitment, gay and bisexual men, human papillomavirus, vaccination promotion, digital intervention, social media, dating apps, recruitment, young adults, adolescents, gay, bisexual, men who have sex with men

## Abstract

**Background:**

Young gay, bisexual, and other men who have sex with men have been referred to as a “hard-to-reach” or “hidden” community in terms of recruiting for research studies. With widespread internet use among this group and young adults in general, web-based avenues represent an important approach for reaching and recruiting members of this community. However, little is known about how participants recruited from various web-based sources may differ from one another.

**Objective:**

This study aimed to determine how young gay, bisexual, and other men who have sex with men recruited from various web-based sources differ from one another in terms of participant characteristics and study engagement.

**Methods:**

Data were collected as part of a randomized controlled trial of Outsmart HPV, a web-based human papillomavirus (HPV) vaccination intervention for young gay, bisexual, and other men who have sex with men. From 2019 to 2021, we recruited young gay, bisexual, and other men who have sex with men in the United States who were aged 18-25 years and not vaccinated against HPV (n=1227) through various web-based avenues. We classified each participant as being recruited from either (1) social media (eg, Facebook, Instagram, Snapchat), (2) a dating app (eg, Grindr, Scruff), or (3) some other digital recruitment source (eg, existing research panel, university-based organization). Analyses compared participants from these 3 groups on demographic and health-related characteristics and metrics involving study engagement.

**Results:**

Most demographic and health-related characteristics differed by web-based recruitment source, including race or ethnicity (*P*<.001), relationship status (*P*<.001), education level (*P*<.001), employment status (*P*<.001), sexual self-identity (*P*<.001), health insurance status (*P*<.001), disclosure of sexual orientation (*P*=.048), and connectedness to the LGBTQ (lesbian, gay, bisexual, transgender, queer) community (*P*<.001) The type of device used by participants during study enrollment also differed across groups, with smartphone use higher among participants recruited via dating apps (n=660, 96.6%) compared to those recruited via social media (n=318, 78.9%) or other digital sources (n=85, 60.3%; *P*<.001). Participants recruited via social media were more likely than those recruited via dating apps to complete follow-up surveys at 3 different timepoints (odds ratios 1.52-2.09, *P*=.001-.008). These participants also spent a longer amount of time viewing intervention content about HPV vaccination (3.14 minutes vs 2.67 minutes; *P*=.02).

**Conclusions:**

We were able to recruit a large national sample of young gay, bisexual, and other men who have sex with men for a web-based HPV vaccination intervention via multiple methodologies. Participants differed on a range of demographic and health-related characteristics, as well as metrics related to study engagement, based on whether they were recruited from social media, a dating app, or some other digital recruitment source. Findings highlight key issues and considerations that can help researchers better plan and customize future web-based recruitment efforts of young gay, bisexual, and other men who have sex with men.

**Trial Registration:**

ClinicalTrials.gov NCT04032106; https://clinicaltrials.gov/study/NCT04032106

**International Registered Report Identifier (IRRID):**

RR2-10.2196/16294

## Introduction

Human papillomavirus (HPV) is the most common sexually transmitted infection in the United States [1], with infections having the potential to cause multiple types of cancer (ie, anal, oropharyngeal, and penile cancers) and genital warts in men [2]. Gay, bisexual, and other men who have sex with men tend to have higher rates of HPV infection and HPV-related disease compared to other men [3-5]. Routine HPV vaccination is currently recommended for those aged 11-12 years in the United States, while also recommended for everyone through the age of 26 years who has not already been vaccinated [6]. However, a recent review paper suggests that fewer than 40% of young gay, bisexual, and other men who have sex with men who are age-eligible for HPV vaccination have received any doses of the vaccine series [5]. In response, HPV vaccination interventions for young gay, bisexual, and other men who have sex with men have recently been developed to improve knowledge and increase vaccination rates [7-10].

One issue central to these interventions is reaching and recruiting young gay, bisexual, and other men who have sex with men as study participants [11]. Gay, bisexual, and other men who have sex with men have previously been referred to as a “hard-to-reach” or “hidden” population for study recruitment [12,13], and traditional recruitment approaches for this community often included snowball sampling, use of “gatekeeper” organizations associated with the community, and venue-based recruitment at community events, health clinics, or other settings [11,14,15]. These traditional approaches faced recruitment challenges including potential participants not being comfortable identifying as gay, bisexual, and other men who have sex with men, lack of inclusive language in recruitment materials, and concerns about stigmatization associated with participation [11-13,15,16].

Web-based recruitment methods, including the use of social media and dating apps, present an alternative approach that has gained popularity for recruiting young gay, bisexual, and other men who have sex with men [17-21]. Social media use is ubiquitous among young adults in the United States, with nearly all reporting prior use [22]. Further, about 80% of young gay, bisexual, and other men who have sex with men report using a dating app on at least a monthly basis [23]. Past studies have shown that digital venues can successfully reach and recruit large numbers of participants, including young gay, bisexual, and other men who have sex with men, and that the content of study advertisements on these platforms can affect recruitment metrics [17,24-29]. Studies have also provided a great deal of information about the costs associated with web-based recruitment [24,28,29] and shown that participants recruited via web-based methodologies are demographically different from those recruited through more traditional approaches [13,30-33]. For example, gay, bisexual, and other men who have sex with men participants recruited via web-based methodologies may differ in terms of age, race or ethnicity, and socioeconomic status (eg, education level) compared to those recruited via more traditional approaches [13,30-32]. Furthermore, a few studies have shown that gay, bisexual, and other men who have sex with men participants tend to be more diverse when multiple recruitment sources are used (eg, using Facebook and Craigslist) [18,21].

Even with our current amount of knowledge about the digital recruitment of young gay, bisexual, and other men who have sex with men, important research gaps remain. One key area to examine is how participants recruited from various web-based sources (eg, social media vs dating apps) may differ from one another. This is true not only for demographic characteristics but also for participants’ study experience (eg, retention, engagement with study content). The latter is particularly relevant to studies that include the delivery of interventions or involve longitudinal data collection. In this report, we address these research gaps by analyzing data from Outsmart HPV, an HPV vaccination intervention study that recruited young gay, bisexual, and other men who have sex with men through various web-based venues. Results will inform and help guide future web-based recruitment efforts of young gay, bisexual, and other men who have sex with men for research studies.

## Methods

### Participants

All data were collected as part of a randomized controlled trial (RCT) of Outsmart HPV. The methods of the RCT have been described previously [7] and are briefly summarized below. We recruited a convenience sample of young gay, bisexual, and other men who have sex with men via social media sites, dating apps, and other web-based avenues. Advertisements on these digital platforms included a combination of images (eg, men in the targeted age range) and brief text about the study (eg, information about HPV, the study being web-based). Although we standardized advertisement content where possible, some content did differ across platforms due to varying advertisement requirements and options (eg, amount of text allowed, advertisement placement). For example, some of our advertisements through dating apps featured content that was sent directly to users’ in-app inboxes over a 24-hour period, which was an approach that was not available across all platforms.

Interested individuals were linked via advertisements to a mobile-friendly project website to complete an eligibility screener. Eligibility criteria included self-identifying as (1) cisgender male; (2) aged 18-25 years; (3) either gay, bisexual, or queer; ever having oral or anal sex with another male; or being sexually attracted to other males; (4) living in the United States; (5) not having received any doses of HPV vaccine; and (5) not having previously participated in Outsmart HPV. Eligible individuals provided informed consent and created a project website account.

Participants next completed a baseline survey (“T1 survey”) and were then immediately randomized using a 1:1:1 allocation scheme to 1 of 3 study groups. The 3 study groups included 2 groups that received intervention content and 1 control group [7]. Participants then viewed intervention content about HPV vaccination on the project website, with participants in both intervention groups viewing Outsmart HPV content about HPV vaccination and participants in the control group viewing standard information about HPV vaccination. After viewing the intervention content, participants completed a second survey (“T2 survey”). Additional follow-up surveys occurred 3 and 9 months later (“T3 survey” and “T4 survey,” respectively). All study surveys were completed on the project website. A total of 1227 participants were randomized from October 2019 to June 2021.

### Measures

The main independent variable for this report was the type of web-based recruitment source, with each participant categorized as being recruited from either: (1) social media (eg, Facebook, Instagram, Snapchat); (2) a dating app (eg, Grindr, Scruff); or (3) some other recruitment source (eg, existing research panel, university-based organization).

The T1 survey collected information on a range of demographic and health-related characteristics. This included participants’ disclosure of their sexual orientation (3 items; *α*=.77; possible range 1-5) [34], concealment of their sexual orientation (3 items; *α*=.75; possible range 1-5) [34], connectedness to the LGBTQ (lesbian, gay, bisexual, transgender, queer) community (2 items; *α*=.72; possible range 1-4), and electronic health literacy (4 items; *α*=.83; possible range 1-5) [35]. Items were coded so that higher values indicate greater levels of a given construct. We examined the type of device that participants used during study enrollment, with each participant categorized as using either a smartphone, tablet device, or a personal computer (eg, desktop, laptop).

To examine study retention, we determined whether participants completed the T2, T3, and T4 surveys. For each survey separately, participants were categorized as having either completed the survey or not completed the survey. Among participants in the 2 intervention groups, we also examined additional metrics of study engagement by calculating the total amount of time (in minutes) spent viewing Outsmart HPV content about HPV vaccination and the total number of logins to the project website. Both of these metrics were winsorized so that the top 10% of values were set to equal the value corresponding to the 90th percentile.

### Data Analysis

We first calculated descriptive statistics for all variables. We examined differences by recruitment source in participants’ demographic and health-related characteristics and device type. To make these comparisons, we used the Pearson chi-square test for categorical dependent variables and 1-way ANOVA (analysis of variance) for continuous dependent variables. We made post hoc pairwise comparisons between the 3 web-based recruitment source groups and used the Bonferroni adjustment to account for multiple comparisons.

We then used regression models to examine differences by recruitment source in study retention and other study engagement metrics. Logistic regression models produced odds ratios (ORs) and 95% CIs for completion of the T2, T3, and T4 surveys. Linear regression models produced standardized β coefficients for the other study engagement metrics. The recruitment source of dating apps served as the referent group in regression models. These statistical tests were 2-tailed with a critical α value of .05. Data were analyzed using R (version 4.2.1; R Foundation for Statistical Computing), and Stata (version 15.0; StataCorp).

### Ethical Considerations

The institutional review board at The Ohio State University approved this study (IRB 2019C0028), and the RCT is registered at ClinicalTrials.gov (NCT04032106). We based the reporting of results for this manuscript on the Strengthening the Reporting of Observational Studies in Epidemiology (STROBE) guidelines [36] since most of the included data were collected at baseline. Informed consent was obtained from all participants, and all data were deidentified. Participants could earn up to US $95 in gift cards for completing study surveys.

## Results

### Participant Characteristics

Overall, 52.8% (n=648) of participants indicated a minoritized racial or ethnic identity, 64.4% (n=790) were between 22 and 25 years of age, 69% (n=847) had at least some college education, and 66.4% (n=815) self-identified as gay. Most participants (n=974, 79.4%) had some form of health insurance, but more than half (n=666, 54.3%) had not had a preventive health visit in the prior year.

A total of 403 (32.8%) participants were recruited via social media, 683 (55.7%) participants via dating apps, and 141 (11.5%) participants via other recruitment sources. Most demographic and health-related characteristics differed by recruitment source (Table 1). Generally, a higher proportion of participants recruited via dating apps reported a minoritized racial or ethnic identity, were single and having sex or casually dating, had less education, reported having sex with a male partner in the past, and had public or no health insurance. Conversely, a higher proportion of participants recruited via social media indicated not being currently employed full-time or part-time, self-identified as gay, and reported being HIV-negative. Participants recruited via social media also reported a higher level of connectedness to the LGBTQ community and a higher level of disclosure of their sexual orientation. Table 1 shows the results of all pairwise comparisons.

The type of device used by participants during study enrollment also differed by recruitment source (*P*<.001; Table 1). Smartphone use was higher among participants recruited via dating apps (n=660, 96.6%) compared to those recruited via social media (n=318, 78.9%) or other web-based sources (n=85, 60.3%). Conversely, personal computer use was higher among participants recruited via social media (n=81, 20.1%) or other web-based sources (n=55, 39%) compared to those recruited via dating apps (n=17, 2.5%).

**Table 1:** Participant characteristics of young gay, bisexual, and other men who have sex with men in the United States by recruitment source for a web-based human papillomavirus vaccination intervention, 2019-2021 (N=1227)^a^.

Characteristics				Social media (n=403)		Dating app (n=683)		Other online sources (n=141)		Test statistic			*P* value	
**Demographic characteristics**														
	**Age (years), n (%)**										1.9			.39
		18-21	144 (35.7)		250 (36.6)		43 (30.5)							
		22-25	259 (64.3)		433 (63.4)		98 (69.5)							
	**Race or ethnicity,** **n (%)**										55.6			<.001^b,c^
		Hispanic	84 (20.8)		244 (35.7)		24 (17)							
		Non-Hispanic White	224 (55.6)		280 (41)		75 (53.2)							
		Non-Hispanic Black	30 (7.4)		85 (12.5)		14 (9.9)							
		Another race or ethnicity	65 (16.1)		74 (10.8)		28 (19.9)							
	**Relationship status,** **n (%)**										50.5			<.001^b,c,d^
		Single and not having sex	57 (14.1)		83 (12.2)		30 (21.3)							
		Single and having sex or casually dating	239 (59.3)		492 (72)		62 (44)							
		In a relationship	107 (26.6)		108 (15.8)		49 (34.8)							
	**Education level,** **n (%)**										38.0			<.001^b,c^
		High school or less	90 (22.3)		261 (38.2)		29 (20.6)							
		Some college or more	313 (77.7)		422 (61.8)		112 (79.4)							
	**Employment status,** **n (%)**										22.9			<.001^b,d^
		Employed full time or part time	253 (62.8)		520 (76.1)		105 (74.5)							
		Other	150 (37.2)		163 (23.9)		36 (25.5)							
	**Region of residence,** **n (%)**										12.1			.06
		Northeast	90 (22.3)		113 (16.5)		25 (17.7)							
		Midwest	77 (19.1)		114 (16.7)		33 (23.4)							
		South	136 (33.8)		245 (35.9)		48 (34)							
		West	100 (24.8)		211 (30.9)		35 (24.8)							
	**Sexual identity, n (%)**										40.2			<.001^b,c,d^
		Gay	296 (73.5)		449 (65.7)		70 (49.7)							
		Bisexual	76 (18.9)		194 (28.4)		47 (33.3)							
		Another identity	31 (7.7)		40 (5.9)		24 (17.0)							
	**Ever had sex with a male, n (%)**										43.3			<.001^b,c,d^
		No	35 (8.7)		24 (3.5)		26 (18.4)							
		Yes	368 (91.3)		659 (96.5)		115 (81.6)							
	**Sexually attracted to males, n (%)**										5.9			.051^c^
		No	12 (3.0)		17 (2.5)		9 (6.4)							
		Yes	391 (97.0)		666 (97.5)		132 (93.6)							
	Connectedness to LGBTQ^e^ community, mean (SD)^f^		2.7 (0.8)		2.4 (0.9)		2.3 (0.8)		29.3			<.001^b,d^		
	Disclosure of sexual orientation, mean (SD)^g^		3.3 (1.6)		3.0 (1.6)		3.1 (1.5)		3.9			.048^b^		
	Concealment of sexual orientation, mean (SD)^h^		2.5 (1.2)		2.7 (1.2)		2.5 (1.1)		0.4			.53		
**Health-related characteristics**														
	**Health insurance, n (%)**										34.1			<.001^b,c^
		Private insurance	285 (70.7)		379 (55.5)		104 (73.8)							
		Public insurance	53 (13.2)		134 (19.6)		19 (13.5)							
		None or do not know	65 (16.1)		170 (24.9)		18 (12.8)							
	**Last preventive health visit, n (%)**								0.7			.72		
		Within the last year	187 (46.4)		306 (44.8)		68 (48.2)							
		More than a year ago	216 (53.6)		377 (55.2)		73 (51.8)							
	**HIV status, n (%)**										7.7			.02^b^
		Negative	392 (97.3)		638 (93.4)		133 (94.3)							
		Positive	11 (2.7)		45 (6.6)		8 (5.7)							
	Electronic health literacy, mean (SD)^i^		4.0 (0.7)		3.9 (0.8)		3.9 (0.8)		2.8			.10		
**Device information**														
	**Device type, n (%)**										175.9			<.001^b,c,d^
		Smartphone	318 (78.9)		660 (96.6)		85 (60.3)							
		Tablet device	4 (1)		6 (0.9)		1 (0.7)							
		Personal computer	81 (20.1)		17 (2.5)		55 (39)							

^a^Due to rounding, percentages may not total 100%. Totals may not equal N=1227 due to missing data. Reported test statistics and *P* values are from Pearson chi-square test for categorical variables and 1-way ANOVA (analysis of variance) for continuous variables. Superscript letters b, c, and d indicate statistically significant differences of post hoc pairwise comparisons using the Bonferroni adjustment to account for multiple comparisons.

^b^Social media compared to dating app.

^c^Dating app compared to other web-based source.

^d^Social media compared to other web-based sources.

^e^LGBTQ: lesbian, gay, bisexual, transgender, queer.

^f^2-item scale; items had a 4-point response scale ranging from 1=“not at all” to 4=“a lot.”

^g^3-item scale; items had a 5-point response scale ranging from 1=“none” to 5=“all.”

^h^3-item scale; items had a 5-point response scale ranging from 1=“never” to 5=“always.”

^i^4-item scale; items had a 5-point response scale ranging from 1=“strongly disagree” to 5=“strongly agree.”

### Study Engagement

**Figure 1:**
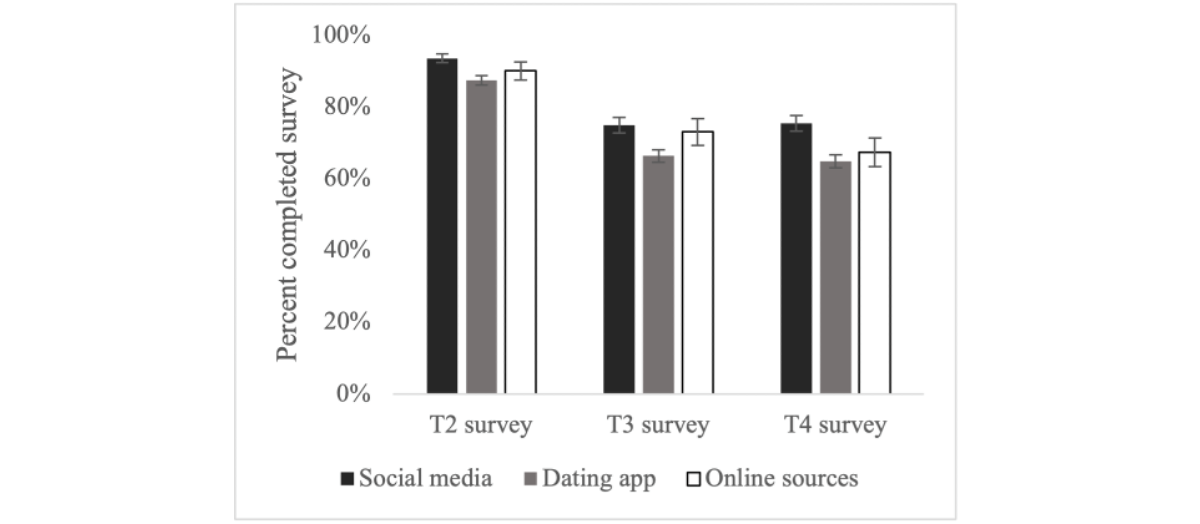
Survey retention of young gay, bisexual, and other men who have sex with men in the United States by recruitment source for a web-based human papillomavirus vaccination intervention (N=1227). Error bars represent SE.

Participant retention overall was 90% for the T2 survey (n=1001), 70% for the T3 survey (n=858), and 69% for the T4 survey (n=842). Compared to participants recruited via dating apps, retention was higher among participants recruited via social media for the T2 survey (94% vs 87%; OR 2.09, 95% CI 1.32-3.30), the T3 survey (75% vs 66%; OR 1.52, 95% CI 1.15-2.00), and the T4 survey (75% vs 65%; OR 1.66, 95% CI 1.26-2.19; Figure 1). There were no statistically significant differences in retention for participants recruited via other web-based sources.

Among participants in the 2 intervention groups (n=815), the average amount of time spent viewing intervention content about HPV vaccination was 2.83 minutes. Participants recruited via social media spent a longer amount of time viewing this content compared to those recruited via dating apps (3.14 minutes vs 2.67 minutes; 95% CI 0.03-0.33; *P*=.02; Table 2). The average number of project website logins was 4.94, and there were no differences in the number of logins by recruitment source (*P*=.83 for social media, *P=*.54 for other web-based sources; Table 2).

**Table 2:** Study engagement of young gay, bisexual, and other men who have sex with men in the United States by recruitment source for a web-based human papillomavirus vaccination intervention (n=815)^a,b^.

	Time spent on intervention content (minutes)			Number of project website log-ins	
	Mean (SD)	β (95% CI)	Mean (SD)		β (95% CI)
Dating app	2.67 (2.44)	Reference	4.98 (2.40)		Reference
Social media	3.14 (2.80)	0.18^b^ (0.03-0.33)	4.94 (2.42)		–0.02 (–0.17 to 0.13)
Other web-based sources	2.74 (2.56)	0.02 (–0.20 to 0.25)	4.81 (2.17)		–0.07 (–0.29 to 0.15)

^a^Table reports data for the 815 participants in the 2 intervention groups. β represents standardized regression coefficients from linear regression models.

^b^Statistically significant at the α=.05 level.

## Discussion

### Principal Results

With digital methodologies becoming increasingly popular for recruiting young gay, bisexual, and other men who have sex with men for research studies, [17-21] it is important to examine if different web-based recruitment sources yield varying types of participants. The Outsmart HPV study provided an opportunity to address this research topic by recruiting a national sample of young gay, bisexual, and other men who have sex with men through multiple web-based sources, and there are 2 main findings. First, most of the demographic and health-related characteristics examined differed by recruitment sources. Participants recruited via social media tended to be less diverse (eg, race or ethnicity, sexual self-identity), of higher socioeconomic status (eg, education level, health insurance status), and have higher levels of outness (eg, disclosure of sexual orientation, connectedness to the LGBTQ community) compared to participants recruited via dating apps. This pattern differs from a past study that found participants recruited from a social media site (Facebook) were highly similar to those recruited from a dating app (Grindr) in terms of race or ethnicity [18]. Interestingly, the characteristics of participants recruited via social media in our study better align with the characteristics of other national samples of young gay, bisexual, and other men who have sex with men and the larger gay, bisexual, and other men who have sex with men community in the United States than participants recruited via dating apps in our study [37-40]. A potential explanation for this pattern is that users of dating apps tend to be more demographically diverse compared to the general population [41]. Interestingly, some characteristics (eg, socioeconomic status) of participants that were recruited via social media for our study were more comparable to gay, bisexual, and other men who have sex with men recruited through in-person venues in past research than via dating apps in our study [42].

Second, study engagement also differed by recruitment source. Participants who were recruited via dating apps tended to interact less with study content and were less likely to complete follow-up surveys compared to participants recruited via social media. This may be attributable to users of dating apps having an emphasis on immediate gratification [43] and impulsivity [44] as a justification for their app usage (and study enrollment), which in turn may affect their awareness of and interest in engaging with study content and completing study-related activities (eg, follow-up surveys). Related, given that dating app users are more impulsive than nonusers, [44] young gay, bisexual, and other men who have sex with men who use dating apps might experience an initial interest in joining a research study and earning an initial study incentive. However, interest in completing study-related activities at later timepoints may not be as strong as their general preference for some level of anonymity [45] might dissuade them from wanting to continue discussing their health behaviors (eg, sexual history, vaccination status).

We think these 2 main findings can help researchers better plan and customize future digital recruitment efforts of young gay, bisexual, and other men who have sex with men by highlighting key issues and considerations. For example, researchers will now be able to better anticipate how the selection of web-based recruitment sources may affect the representativeness and diversity (and the potential balance between the two) of their study sample. Recruiting through social media may lead to a sample of participants that better reflect the larger young gay, bisexual, and other men who have sex with men population in the United States, but dating apps may be more effective in reaching more diverse young gay, bisexual, and other men who have sex with men, including those from communities that are often underrepresented in research studies [46]. Recruitment goals differ across studies in terms of representativeness and diversity, and our data on differences in demographic and health-related characteristics can help inform future efforts. Furthermore, studies involving interventions and/or longitudinal data collection should consider how various web-based recruitment sources can impact study retention and participants’ engagement with study content. Some studies may want to maximize participant retention and engagement (ie, suggesting that recruitment primarily through social media may be appropriate), whereas other studies may be willing to have slightly lower study engagement metrics in exchange for being able to include additional recruitment sources, such as dating apps. Future studies that do include recruitment from dating apps may also want to consider prioritizing brief study-related activities and other strategies to promote engagement.

One final implication involves the types of devices that were used by participants in our study. Overall, a majority of participants used a smartphone to enroll in the study, including nearly all participants who were recruited via dating apps. This pattern is similar to national data showing that young adults use smartphones much more frequently than other device types [47,48]. Thus, it is important that future digital interventions and other research studies for young adults, including young gay, bisexual, and other men who have sex with men, ensure that study content and data collection instruments are optimized for use on smartphones. For example, to increase usability, it has been suggested that the interface of smartphone-based interventions have a minimal number of screens, limited manual data entry, limited pop-ups and notifications, and personal identification number-based entry (as compared to full passwords). The content of such interventions should be succinct with easy-to-read graphics [49]. Data collection instruments (eg, surveys) should have limited open-ended questions and different “pages” in the survey, rather than having participants scroll through one continuous page. Lastly, and perhaps most generally, mobile surveys should be pilot-tested prior to the start of data collection activities [50]. As smartphone usage continues to be a common approach for participants enrolling in and interacting with digital interventions, such recommendations can help optimize intervention delivery and data collection.

### Limitations

Although we were able to compare different groupings of web-based recruitment sources, we were not able to examine potential differences between individual platforms (eg, Facebook vs Twitter vs Grindr) due to modest sample sizes for some of the platforms. These sample sizes also did not allow us to examine how differences between recruitment sources may have changed over time during the recruitment period for our study. Examining potential differences between individual platforms and potential temporal changes are important areas for future research. Our recruitment focused on the social media and dating apps that were among the most popular among young adults in the United States around the time of the start of our study [51,52]. It is likely that the use and popularity of various platforms will evolve over time, and future efforts should examine new and emerging platforms. We were not able to examine advanced metrics related to recruitment efficiency and cost due to a lack of available data. However, past studies have reported such information for various web-based platforms [17,27]. Study recruitment occurred both prior to and during the COVID-19 pandemic, and it is not known how the pandemic may have affected the interest of young gay, bisexual, and other men who have sex with men to enroll in a study focusing on vaccination (which would subsequently affect the characteristics of study participants). Fraudulent accounts with inaccurate self-reported data occur in digital studies, but we used several recommended strategies for detecting such accounts and minimizing this risk [53,54].

### Conclusions

We were able to recruit a large national sample of young gay, bisexual, and other men who have sex with men for an HPV vaccination intervention via multiple web-based methodologies. Participants differed on a range of demographic and health-related characteristics based on whether they were recruited from social media, a dating app, or some other web-based recruitment source. Study retention and engagement with study content also differed by web-based recruitment source. These findings and patterns highlight key issues and potential tradeoffs that can help researchers better plan and customize future web-based recruitment efforts of young gay, bisexual, and other men who have sex with men.

## Abbreviations

ANOVA: analysis of variance
HPV: human papillomavirus
LGBTQ: lesbian, gay, bisexual, transgender, and queer
RCT: randomized controlled trial
STROBE: Strengthening the Reporting of Observational Studies in Epidemiology

## Data Availability

The data for this study may be made available from the corresponding author on reasonable request.
